# Supplementary material for: A new experimental evidence-weighted signaling pathway resource in FlyBase
Source: Development. 2024 Feb 5;151(3):dev202255. doi: 10.1242/dev.202255 (PMC10911275; doi:10.1242/dev.202255)
Supplement: Supplementary information [file develop-151-202255-s1.pdf]

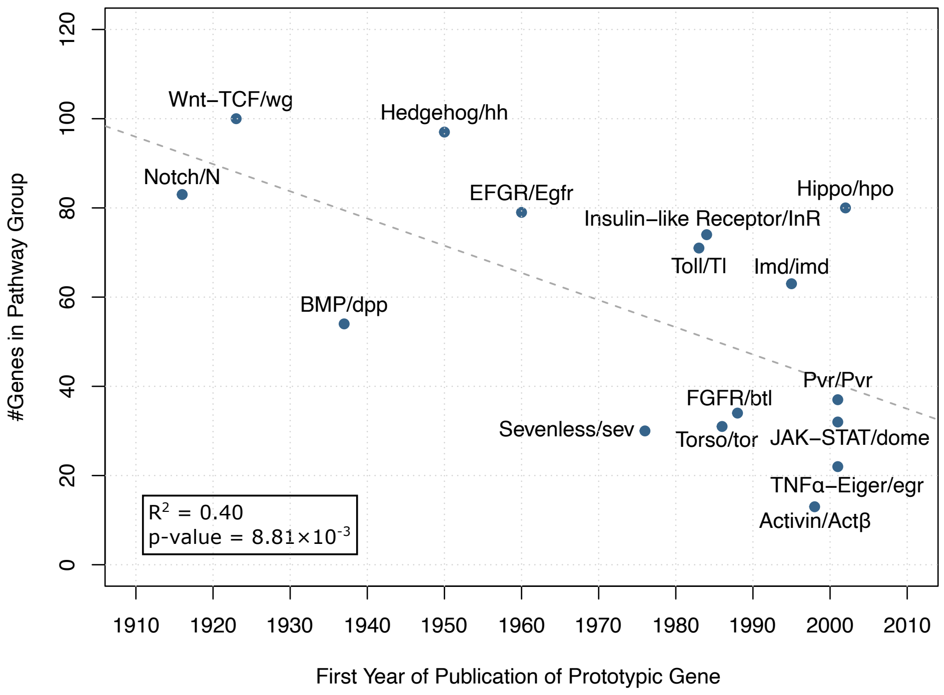

Fig. S1. Pathways studied for a greater length of time do not necessarily have more identified components. The graph plots the year the prototypic member was first characterized, as measured by the first research paper associated with the gene in FlyBase, versus the number of curated pathway members. Linear regression was performed using the *lm* function in R.

Export | Other available columns: # All Research Refs, Also Known As, Antibody, Classical / Insertion Alleles, Transgenic constructs, Disease Models (Experimental), Potential Disease Models, Other Pathways, Human orthologs | Show/Hide Columns

All 10 100 | « 1 2 3 »

Gene Symbol	Gene Name	Gene Group Membership	GO Molecular Function (Experimental)	# Pathway Refs
Filter...	Filter...	Filter...	Filter...	Filter...
Egfr	Epidermal growth factor receptor	RECEPTOR TYROSINE KINASES	epidermal growth factor receptor activity	16
spi	spitz	EGFR AGONISTS	epidermal growth factor receptor binding<br>receptor ligand activity	15
rl	rolled	CONVENTIONAL MITOGEN ACTIVATED PROTEIN KINASES	DNA-binding transcription factor binding<br>MAP kinase activity<br>protein kinase binding<br>protein serine/threonine kinase activity	10
Ras85D	Ras oncogene at 85D	RAS GTPASES	G protein activity<br>GTPase activity	8
vn	vein	EGFR AGONISTS	epidermal growth factor receptor binding<br>heparin binding<br>receptor ligand activity	7
Raf	Raf oncogene	NON-RECEPTOR TKL KINASES	kinase binding<br>MAP kinase kinase kinase activity<br>protein serine/threonine kinase activity	6
grk	gurken	EGFR AGONISTS	epidermal growth factor receptor binding<br>receptor ligand activity	6
cnk	connector enhancer of ksr		enzyme regulator activity<br>MAP-kinase scaffold activity<br>mitogen-activated protein kinase kinase kinase binding<br>receptor tyrosine kinase	5

Fig. S2. EGFR Signaling Pathway Core Components Members Table. A screenshot of the EGFR Signaling Pathway Core Components (FBgg0000951) members table showing the default column view sorted by # pathway refs (i.e. supporting papers for that gene annotated with 'epidermal growth factor receptor signaling pathway' (GO:0007173) using an experimental evidence code).

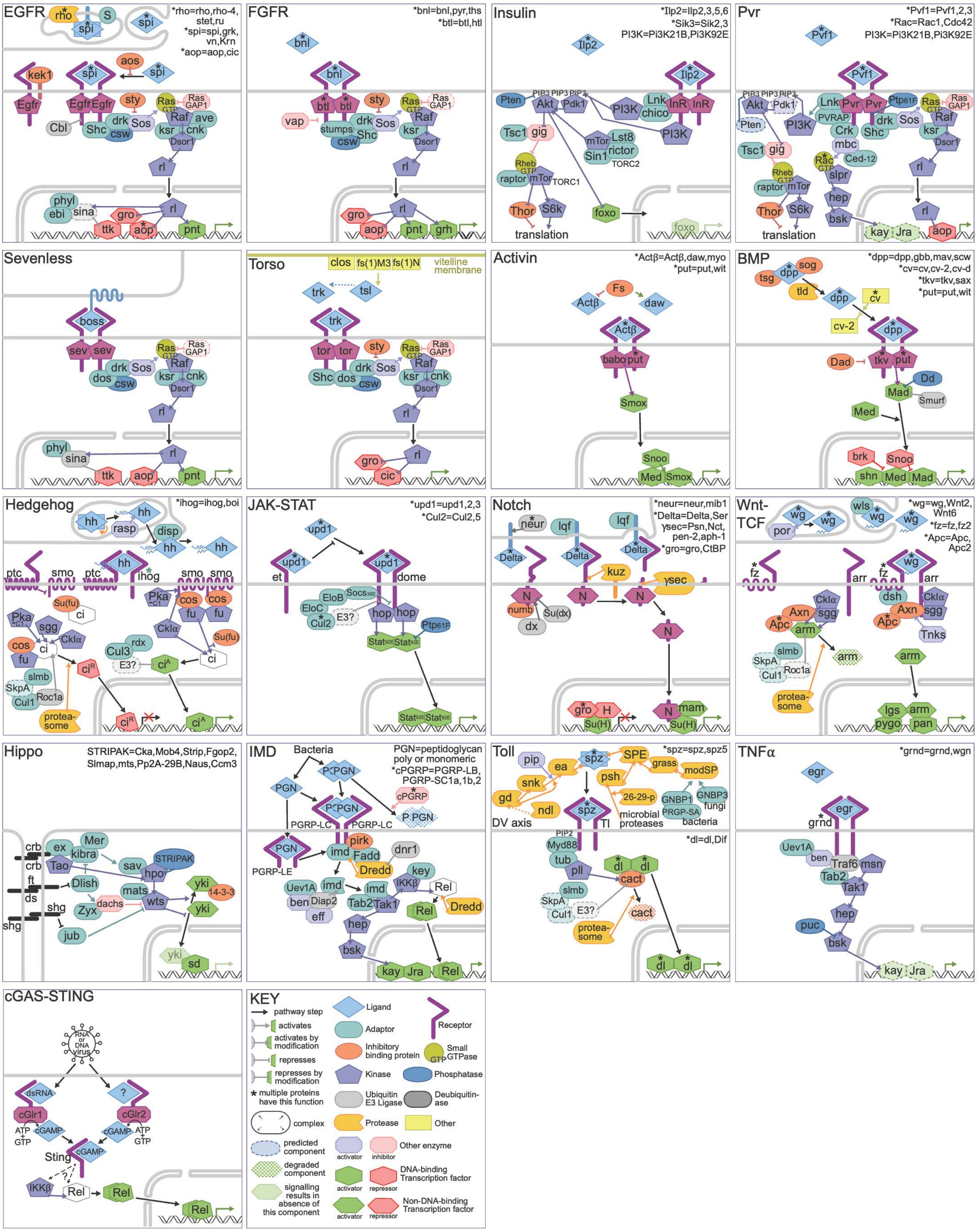

Fig. S3. Thumbnails of signaling pathways in the FlyBase resource. The 17 pathways detailed in this paper that have been reviewed by evidence-weighted curation are presented in FlyBase. For each, a text-book style over-view is available. In a few instances, a key component lacks experimental evidence in *Drosophila* and so has been added as a 'predicted component' with a dashed-line border. These Thumbnail images were created in Adobe illustrator. The Pathway thumbnails were reviewed by experts in the field (see Acknowledgments).

Development • Supplementary information

Export ▾ Other available columns: Gene Group Membership, GO Molecular Function (Experimental), # All Research Refs, Also Known As, Antibody, Classical / Insertion Alleles, Transgenic constructs, Other Pathways, Human orthologs Show/Hide Columns

All 10 100 « 1 2 »

Gene Symbol	Gene Name	# Pathway Refs ↓	Disease Models (Experimental)	Potential Disease Models
Filter...	Filter...	Filter...	Filter...	Filter...
tkv	thickveins	24	juvenile polyposis syndrome<br>juvenile polyposis syndrome	acromesomelic dysplasia-3<br>brachydactyly type A1D<br>brachydactyly type A2<br>hereditary mixed polyposis syndrome 2<br>juvenile polyposis syndrome
dpp	decapentaplegic	22	juvenile polyposis syndrome	brachydactyly type A2<br>hemochromatosis type 1<br>orofacial cleft 11<br>syndromic microphthalmia 6
Mad	Mothers against dpp	20	intestinal cancer<br>juvenile polyposis syndrome	primary pulmonary hypertension
put	punt	13	juvenile polyposis syndrome	esophageal cancer<br>hereditary nonpolyposis colorectal cancer type 6<br>Loeys-Dietz syndrome 2<br>persistent Mullerian duct syndrome<br>visceral heterotaxy
sax	saxophone	13	fibrodysplasia ossificans progressiva	fibrodysplasia ossificans progressiva<br>hereditary hemorrhagic telangiectasia
Med	Medea	13	colorectal cancer<br>intestinal cancer<br>juvenile polyposis syndrome	juvenile polyposis syndrome<br>juvenile polyposis-hereditary hemorrhagic telangiectasia syndrome<br>pancreatic carcinoma

Fig. S4. Disease associations for members of the BMP Signaling Pathway Core Components page. Columns showing experimental and predicted (computed from human-ortholog OMIM links) show that six members of the BMP Signaling Pathway Core Components (FBgg0001085) have been used to model ‘juvenile polyposis syndrome’ (DOID:0050787) in flies and two members have human orthologs with ‘juvenile polyposis syndrome’ associations in OMIM.

Table S1. Summary of Signaling Pathways content in FlyBase.

An Excel spreadsheet summary of the Signaling Pathway page content in FlyBase release version FB_2023_01. The name and unique FlyBase Gene Group ID (FBgg#) are given in the first column. The second column is the GO term and ID that is mapped to the FBgg. The number of genes and papers linked to the pathway are given into the last two columns. Note that the sum of the numbers for the member groups is often different than for the top-level group as papers and genes may be shared between more than 1 subgroup. For each pathway group, except cGAS/STING, the table also includes the gene selected as the prototypic representative and year of publication of first research paper on the prototype.

Available for download at
https://journals.biologists.com/dev/article-lookup/doi/10.1242/dev.202255#supplementary-data
